# Case Report: Neonatal Complex Congenital Heart Disease With Anomalous Origin of the Left Coronary Artery From the Right Pulmonary Artery: Analysis of Missed Diagnosis and Improvement Procedures

**DOI:** 10.3389/fped.2021.805632

**Published:** 2022-01-28

**Authors:** Zhen Bing, Leilei Liu, Rui Chen, Qian Cao, Wen Ge

**Affiliations:** ^1^Heart Center, Qingdao Women and Children's Hospital, Affiliated to Qingdao University, Qingdao, China; ^2^Department of General Medicine, Qingdao Women and Children's Hospital, Affiliated to Qingdao University, Qingdao, China; ^3^Radiology Department, Qingdao Women and Children's Hospital, Affiliated to Qingdao University, Qingdao, China

**Keywords:** neonate, congenital heart disease, anomalous origin of the left coronary artery from the right pulmonary artery, missed diagnosis, improvement procedure

## Abstract

The anomalous origin of the left coronary artery from the right pulmonary artery is a rare type of congenital disease. It is even rarer when combined with complex congenital heart diseases requiring surgical intervention in the neonatal period. Because it has no clinical manifestations in the neonatal period, it is easier to miss diagnosis when combined with complex congenital heart disease. To avoid a missed diagnosis of anomalous origin of the left coronary artery from the right pulmonary artery, preoperative echocardiography should routinely explore the orifice of the coronary artery. However, the preoperative examination can lead to missed diagnosis due to the influence of the examiner's experience, equipment, and other factors. After thoracotomy, exploring the orifice position of the left and right coronary arteries can avoid a missed diagnosis of the abnormal origin of coronary arteries. An exploration of the coronary artery is mainly recommended for children with complex congenital heart disease in the neonatal period and children with congenital heart disease combined with unexplained cardiac insufficiency and abnormal mitral valve development.

## Introduction

The anomalous origin of the left coronary artery from the right pulmonary artery (ALCAPA) is a rare type of congenital disease. Combined with complex congenital heart diseases (CHDs) requiring surgical intervention in the neonatal period, it is even rarer (1–3). We report a neonate with complex CHD. It was diagnosed as coarctation of the aorta with arch hypoplasia, ventricular septal defect (VSD), atrial septal defect (ASD), and patent ductus arteriosus (PDA). The preoperative examination missed the diagnosis of the ALCAPA. After the operation, it was impossible to get out of cardiopulmonary bypass due to severe left cardiac insufficiency, which eventually led to death. This report focuses on analyzing the causes of missed diagnosis and formulating improvement procedures to avoid recurrence.

## Case Presentation

**Figure 1:**
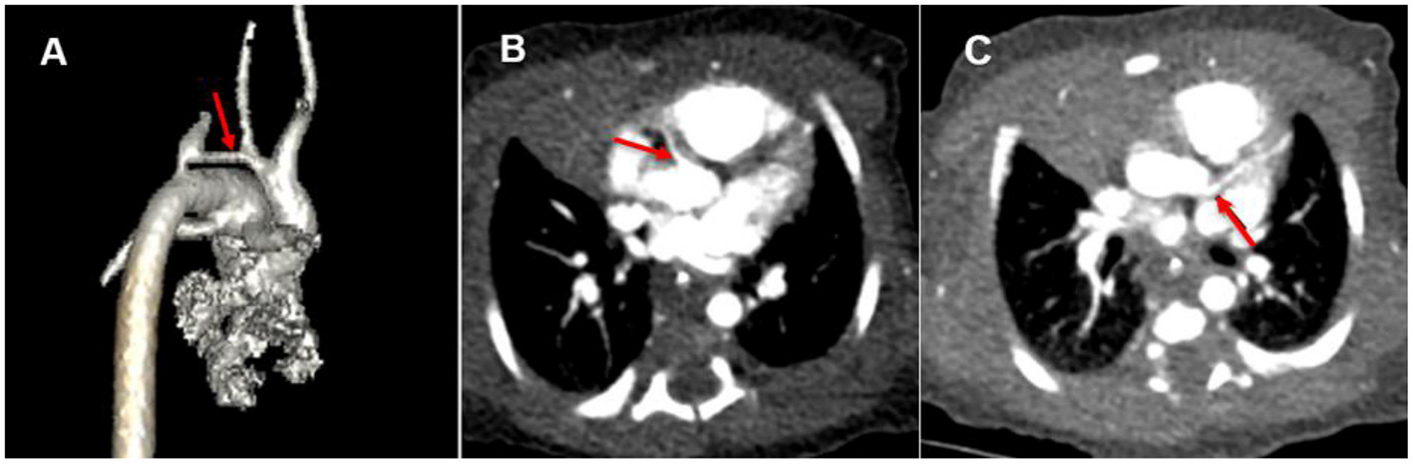
**(A)** Coarctation of the aorta with severe tubular arch hypoplasia (red arrow). **(B)** Origin of right coronary artery (red arrow). **(C)** False signs of left coronary artery origin from aorta (red arrow).

A female infant (38 weeks, 3.35 kg) was diagnosed with coarctation of the aorta with severe tubular arch hypoplasia and VSD by prenatal ultrasound. Transcutaneous oxygen saturation was 95% (room air) on the second day after birth. Echocardiography showed that in the coarctation of the aorta with severe tubular arch hypoplasia, the inner diameter of the arch was about 2.3 mm, the length was about 11 mm, and the blood flow was less. The VSD diameter was 5.4 mm, and the ASD diameter was 3 mm. The inner diameter of the pulmonary end of PDA was 5.3 mm, with a right-to-left shunt. The pulmonary arterial systolic pressure was 63 mmHg. No sign of the ALCAPA was found, and the valve was normal. Left ventricular ejection fraction (LVEF) was 64%. The newborn was transferred to intensive care unit (ICU). The myocardial enzyme and electrocardiogram (ECG) were normal. Lower limb transcutaneous oxygen saturation decreased on the second day after birth and improved after prostaglandin E treatment. Computed tomography angiography (CTA) was done to evaluate aortic arch dysplasia (Figure 1A), and there were no obvious abnormalities in the origin and course of left and right coronary arteries (Figures 1B,C).

**Figure 2:**
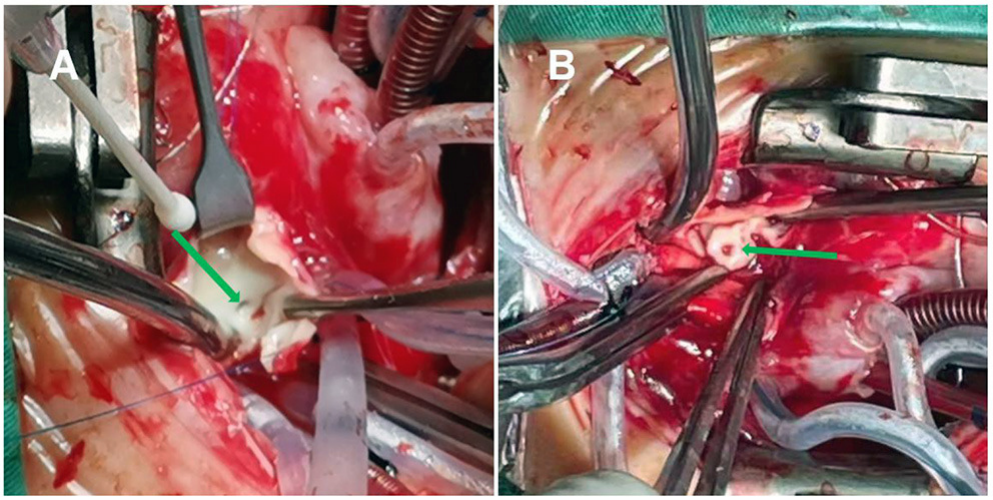
**(A)** The opening of the left pulmonary artery in the posterolateral wall at the beginning of the right pulmonary artery (green arrow). **(B)** The left coronary artery was buttoned off from the pulmonary artery wall (green arrow).

On the 13th day after birth, under general anesthesia and hypothermic cardiopulmonary bypass, the children underwent a correction of the coarctation of the aorta with severe tubular arch hypoplasia, a repair of the VSD, a repair of the ASD, and a closure of the PDA. Cardiopulmonary bypass was established after median thoracotomy. The circulation was blocked; the cardioplegia solution was perfused through the aortic root, and then the heart stopped completely. The VSD was repaired, and the PDA was cut and sutured. Selective cerebral perfusion was performed, and there was no circulatory arrest. The small bend side of the aortic arch was anastomosed with the end side of the descending aorta. Finally, the ASD was repaired. The operation was completed. The aortic cross-clamping time was 100 min. After fully exhausting the gas, the block was opened for systemic perfusion. The heart beat again slowly, and it was difficult to maintain the blood pressure after reducing the flow. Transesophageal echocardiography showed severe left ventricular dysfunction. Surgical exploration was performed again. The origin and course of the right coronary artery were normal. The main trunk of the left coronary artery was located behind the main pulmonary artery and ran through the atrioventricular sulcus. The absence of the left coronary artery originating from the aorta was confirmed by gently pulling up the aorta and pressing down the right atrial appendage, which indicated the suspected ALCAPA. The aorta was blocked again, and the cardioplegia solution was perfused. It was found that the left coronary artery was poorly filled. The orifice of the left coronary artery at the beginning of the right pulmonary artery could be seen by an incision of the pulmonary artery (Figure 2A). The cardioplegia solution was also perfused into the left coronary artery. The left coronary artery was buttoned off from the pulmonary artery wall (Figure 2B), anastomosed to the corresponding position of the aorta without tension and distortion, and the right pulmonary artery and main pulmonary artery were repaired with an autologous pericardial patch. The second aortic cross-clamping time was 58 min. After reopening and blocking, the left coronary artery was well filled. However, the recovery of left ventricular function was poor. Many attempts to wean from bypass were not possible due to unknown ALCAPA. Extracorporeal membrane oxygenation adjuvant therapy was planned, but the parents refused. After prolonged cardiopulmonary bypass time, the left ventricular function did not improve, and the heart rate and blood pressure decreased. Vasoactive drugs could not be maintained, and the patient eventually died.

## Discussion

**Figure 3:**
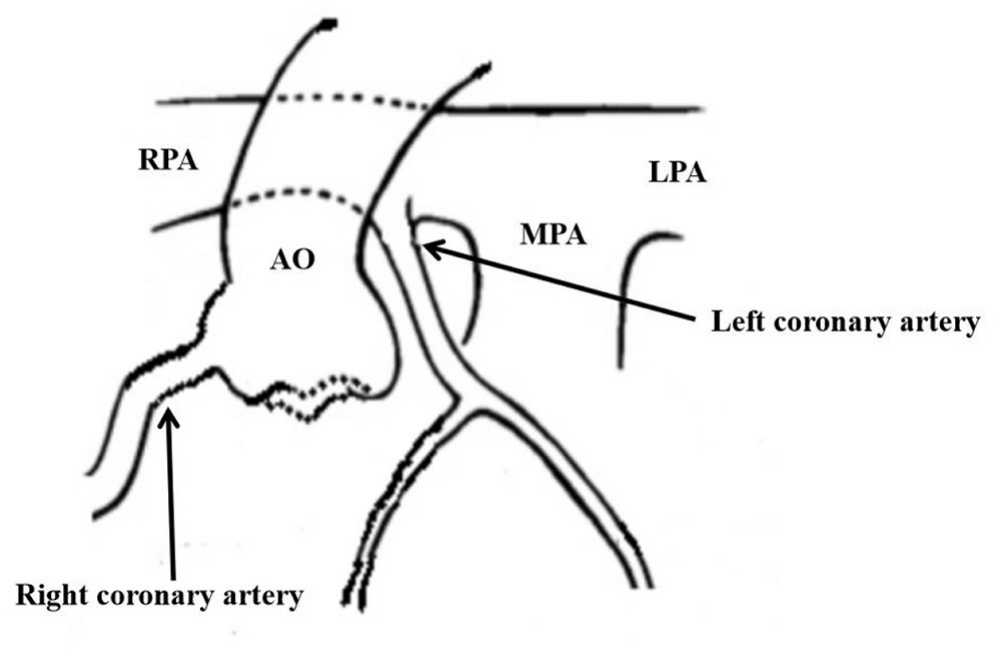
The abnormality of left coronary artery originated from the posterolateral wall at the beginning of right pulmonary artery.

This case reports an extremely rare disease that the anomalous origin of the left coronary artery from the posterolateral wall of the right pulmonary artery (Figure 3) complicated with complex CHD in the neonatal period. In the early stage of a newborn, most children with the ALCAPA have no clinical symptoms. ECG can have no abnormal changes in Q wave and the ST-T segment, which is easy to be missed (4, 5). In the neonatal period of complex CHD, if the diagnosis of ALCAPA is missing, the myocardium in the blood supply area of the left coronary artery cannot be protected after a cardioplegia solution is perfused at the root of the aorta. If the operation lasts for a long time, it can lead to serious left ventricular dysfunction and even the heart cannot rebound (6).

Echocardiography is an important tool for diagnosing the ALCAPA. The direct signs include that the left coronary artery is not connected with the left coronary sinus of the aorta, but with the pulmonary artery, and the blood flow of the left coronary artery retrogradely enters the pulmonary artery in diastole. Indirect signs include compensatory dilation of the right coronary artery, the formation of left and right coronary collateral circulation in the myocardium, left ventricular enlargement, mitral insufficiency, and endocardial elastic fiber hyperplasia (7, 8). In the neonatal period, due to the existence of pulmonary hypertension, the blood supply of the ALCAPA mainly or completely comes from the pulmonary artery (8). If combined with PDA or VSD, the pulmonary artery pressure and pulmonary artery oxygen partial pressure will not be significantly reduced, with no secondary changes of myocardial ischemia, and the collateral circulation of the coronary artery will not be formed (9). In this situation, echocardiography can only see the abnormal vascular opening of the pulmonary artery, while there is no opening of the left coronary artery of the aorta (10). The abnormal opening of the left coronary artery can be located at any part of the pulmonary artery. The most common location is the left posterior sinus at the root of the pulmonary artery, followed by the right posterior sinus, the posterior wall of the main pulmonary artery, and the posterior side of the beginning of the right pulmonary artery (11). The abnormal origin of the left coronary artery mostly originates from the left posterior sinus at the root of the pulmonary artery, and its trunk is far away from the main pulmonary septum. However, in rare cases, the abnormal coronary artery can originate from the right wall of the main pulmonary artery or the beginning of the right pulmonary artery, and the opening is closely adjacent to the left coronary sinus of the aorta. In addition, the left main coronary artery runs normally, which easily gives a false impression of the normal origin of the coronary artery on echocardiography (12). Especially when combined with complicated CHDs, the diagnostic focus is shifted to other significant cardiac malformations, easily resulting in a missed diagnosis.

CTA can directly display the location of the abnormal origin of the coronary artery and the abnormal density of the contrast medium in the main pulmonary artery. However, CTA has requirements for imaging conditions. ECG-gated axial scan, which can freeze the heartbeat and well show the indirect changes associated with coronary artery origin, coronary artery dilatation, internal and external collateral vessels, myocardial ischemia, cardiac cavity enlargement, and valve involvement, has a high diagnostic value (13). However, due to the rapid heart rate, the newborn above was not suitable for an ECG-gated axial scan. A spiral scan could only be used. It was sensitive to the heartbeat and difficult to show the origin of the coronary artery. In addition, due to severe pulmonary hypertension and extensive traffic of contrast medium through VSD, ASD, and PDA, there was no abnormal density change of contrast medium in the pulmonary artery. More importantly, the abnormal left coronary artery, in this case, originated from the posterolateral wall at the beginning of the right pulmonary artery, which was adjacent to the left coronary sinus of the aorta. The heart rate and respiration of the newborn with critical CHD were significantly faster, the respiratory amplitude was also increased, the application of ECG gating was limited, and artifacts were generated during coronary imaging, resulting in the illusion that the left coronary artery originated from the aorta (Figure 1C).

Neonatal complex CHD complicated with the ALCAPA is a rare and easy-to-miss diagnosis, which can lead to serious adverse events. Therefore, the origin of the coronary artery should be routinely detected by preoperative echocardiography in neonatal complex CHD. However, missed diagnosis may still occur due to the complexity of the disease, the inspector's experience, instruments, and other factors. After thoracotomy, the key step to avoid a missed diagnosis of the abnormal origin of the coronary artery is to explore the origin of the left and right coronary arteries firstly. The filling degree of left and right coronary arteries should be paid attention to when cardioplegia fluid is perfused by aortic intubation. If severe left ventricular dysfunction occurs after the operation, it is necessary to be vigilant and look for evidence of the ALCAPA. An exploration of the coronary artery is mainly recommended for children with complex CHD in the neonatal period and children with CHD with unexplained cardiac insufficiency and abnormal mitral valve development, which may be indirect signs of the ALCAPA (1, 7, 8). The above improvement procedures are strongly recommended in neonatal complex CHD surgery, which can avoid the missing diagnosis of the ALCAPA.

## Data Availability Statement

The original contributions presented in the study are included in the article/Supplementary Material, further inquiries can be directed to the corresponding author.

## Ethics Statement

Written informed consent was obtained from the parents of the participant for the publication of any potentially identifiable images or data included in this article.

## Author Contributions

ZB is responsible for drafting the manuscript and approving the final version to be published. LL participated in the writing and revision of the paper. RC directed the writing of the entire article, especially the surgical procedure. QC participates in the revision of the paper, especially the content related to extracorporeal circulation. WG participated in the revision of imaging-related content in the paper. All authors read and approved the final manuscript.

## Conflict of Interest

The authors declare that the research was conducted in the absence of any commercial or financial relationships that could be construed as a potential conflict of interest.

## Publisher's Note

All claims expressed in this article are solely those of the authors and do not necessarily represent those of their affiliated organizations, or those of the publisher, the editors and the reviewers. Any product that may be evaluated in this article, or claim that may be made by its manufacturer, is not guaranteed or endorsed by the publisher.

## References

[1] Triglia LT Guariento A Zanotto L Zanotto L Cattapan C Hu R. Anomalous left coronary artery from pulmonary artery repair: Outcomes from the European Congenital Heart Surgeons Association Database. J Card Surg. (2021) 36:1910–6. 10.1111/jocs.15448 33651393

[2] Vollroth M Hambsch J Daehnert I Gebauer R Riede FT Bakhtiary F. Anomalous origin of the left coronary artery from the right pulmonary artery: an extremely rare cardiac malformation. Ann Thorac Surg. (2013) 96:e21. 10.1016/j.athoracsur.2013.03.068 23816112

[3] Binsalamah ZM Lara DA McKenzie ED. Anomalous origin of the left coronary artery from the right pulmonary artery in a univentricular heart. Cardiol Young. (2017) 9:1853–6. 10.1017/S1047951117001317 28651676

[4] Walker TC Renno MS Parra DA Guthrie SO. Neonatal ventricular fibrillation and an elusive ALCAPA: things are not always as they seem. BMJ Case Rep. (2016) 2016:bcr2015214239. 10.1136/bcr-2015-214239 27033289 PMC4840740

[5] Celik L Becker V Hammel D Nürnberg JH. Early detection of anomalous origin of left coronary artery from the right pulmonary artery after successful repair of critical coarctation of the aorta. Pediatr Cardiol. (2010) 31:294–6. 10.1007/s00246-009-9595-y 19960190

[6] Reddy SR Herbert CE Zellers TM. ACCAPA anomalous circumflex coronary artery origin from pulmonary artery. Cardiol Young. (2020) 30:1730–1. 10.1017/S1047951120003352 33203499

[7] Lin S Xie M Lv Q Wang J He L Wang B. Misdiagnosis of anomalous origin of the left coronary artery from the pulmonary artery by echocardiography: Single-center experience from China. Echocardiography. (2020) 37:104–13. 10.1111/echo.14578 31981242

[8] Memon MK Muneer Amanullah MA. Anomalous left coronary artery from pulmonary artery: an important cause of ischemic mitral regurgitation in children. Cureus. (2019) 11:4441. 10.7759/cureus.4441 31245227 PMC6559683

[9] Korkmaz AA Erkan H Karadeniz A Orem C. Missed diagnosis and treatment dilemma: large patent ductus arteriosus combined with anomalous origin of the left coronary artery from the pulmonary artery. Iran J Radiol. (2017) 2:1–5. 10.5812/iranjradiol.30713

[10] Grimaldi A Ammirati E La Canna G Sora N Faletra F De Bonis M. Echocardiographic 'brainstorm' to detect anomalous origin of the left coronary artery from the pulmonary artery. J Cardiovasc Med. (2012) 13:152–5. 10.2459/JCM.0b013e328343cc47 21430548

[11] Dodge-Khatami A Mavroudis C Backer CL. Anomalous origin of the left coronary artery from the pulmonary artery: collective review of surgical therapy. Ann Thorac Surg. (2002) 74:946–55. 10.1016/S0003-4975(02)03633-0 12238882

[12] Wang SS Chen XX Chen JM Zhang ZW Ma Y Huang MP. Echocardiographic findings of an anomalous origin of the left coronary artery in children and adolescents: real or fake? J Ultrasound Med. (2016) 35:1783–90. 10.7863/ultra.15.11019 27353070

[13] Ojha V Pandey NN Kumar S Ramakrishnan S Jagia P. Anomalous origin of left main coronary artery from pulmonary artery: patient characteristics and imaging associations on multidetector computed tomography angiography. J Card Surg. (2021) 36:4043–53. 10.1111/jocs.15926 34414605

