# Supplementary figures and images for: Case Report: Neonatal Complex Congenital Heart Disease With Anomalous Origin of the Left Coronary Artery From the Right Pulmonary Artery: Analysis of Missed Diagnosis and Improvement Procedures
Source: Front Pediatr. 2022 Jan 28;9:805632. doi: 10.3389/fped.2021.805632 (PMC8833846; doi:10.3389/fped.2021.805632)

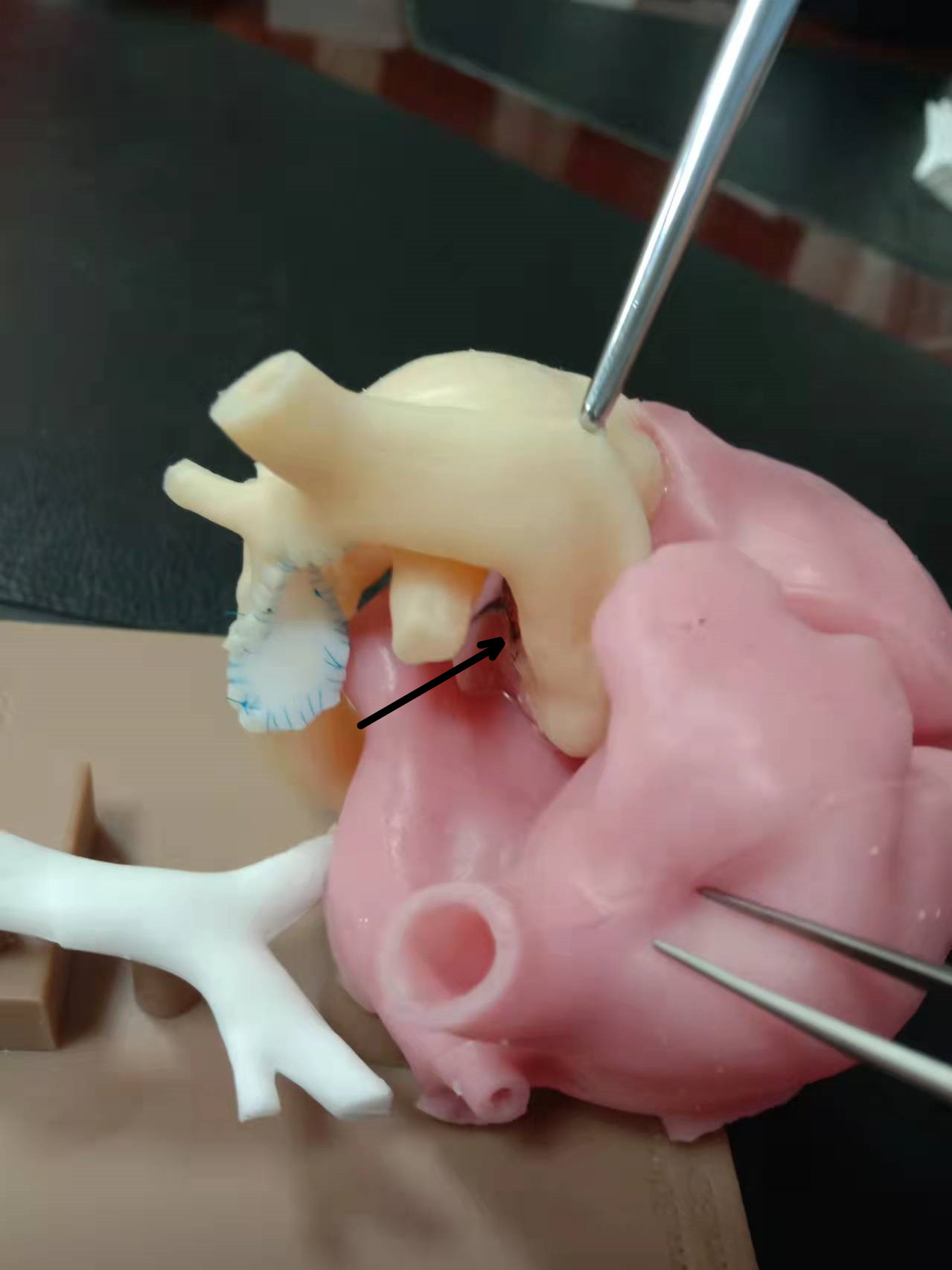

Supplement: Supplementary file 1 [file Image_1.JPEG]
